# Effects of Rosmarinic Acid and Sinapic Acid on the Skeletal System in Ovariectomized Rats

**DOI:** 10.3390/nu18020301

**Published:** 2026-01-18

**Authors:** Maria Zych, Radosław Wolan, Agnieszka Włodarczyk, Piotr Londzin, Weronika Borymska, Ilona Kaczmarczyk-Żebrowska, Joanna Folwarczna

**Affiliations:** 1 Department of Pharmacognosy and Phytochemistry, Faculty of Pharmaceutical Sciences in Sosnowiec, Medical University of Silesia, Katowice, Jagiellońska 4, 41-200 Sosnowiec, Poland; weronika.borymska@sum.edu.pl (W.B.); izebrowska@sum.edu.pl (I.K.-Ż.); 2 Department of Pharmacology, Faculty of Pharmaceutical Sciences in Sosnowiec, Medical University of Silesia, Katowice, Jagiellońska 4, 41-200 Sosnowiec, Poland; radoslaw.wolan@sum.edu.pl (R.W.); piotr.londzin@vp.pl (P.L.); 3 Department of Medical Biophysics, Faculty of Medical Sciences in Katowice, Medical University of Silesia, Katowice, Medyków 14, 40-752 Katowice, Poland; awlodarczyk@sum.edu.pl

**Keywords:** rosmarinic acid, sinapic acid, estrogen deficiency, osteoporosis

## Abstract

**Background/Objectives**: It is believed that some polyphenols, including phenolic acids, may counteract estrogen deficiency-induced bone loss, decreasing oxidative stress. Moreover, some phenolic acids—among others, rosmarinic acid and sinapic acid—have been reported to increase the serum estradiol concentration in rats. The study aimed to investigate the impact of rosmarinic acid and sinapic acid on the skeletal system of rats with estrogen deficiency induced by bilateral ovariectomy. **Methods**: The study was carried out on mature female rats, divided into sham-operated control rats, ovariectomized (OVX) control rats, and OVX rats treated with estradiol (0.2 mg/kg; positive control), rosmarinic acid (10 and 50 mg/kg), or sinapic acid (5 and 25 mg/kg). The compounds were administered orally for 4 weeks. Serum bone turnover markers, bone mass, mineral and calcium content, macrometric and histomorphometric parameters, as well as mechanical properties were examined. **Results**: Estrogen deficiency induced osteoporotic changes in ovariectomized control rats, which were slightly counteracted by the administration of estradiol. The phenolic acids slightly counteracted some changes caused by estrogen deficiency, but their administration at higher doses led to further worsening of cancellous bone quality. **Conclusions**: The results demonstrated that administration of high doses of rosmarinic acid or sinapic acid slightly unfavorably affected the rats’ skeletal system under conditions of estrogen deficiency.

## 1. Introduction

Osteoporosis is a chronic bone disease characterized by a decrease in bone mass, impairment of bone microarchitecture, and a reduction in bone mineral density. These changes lead to a deterioration in bone mechanical properties and an increased risk of fractures, which, in some cases, result in complete loss of mobility or permanent disability [1]. Due to the aging population and the increasing prevalence of osteoporosis, the condition is considered a significant public health issue in many developed countries [2]. Osteoporosis most commonly affects women during the postmenopausal period, which is linked to a decrease in estrogen levels, as estrogens play a crucial role in the modulation of bone metabolism [3]. Other conditions, including diabetes and chronic kidney disease, which become more prevalent with age, may also contribute to the development of osteoporosis [4].

In the pathogenesis of osteoporosis, oxidative stress and the associated inflammatory responses may play a crucial role. These factors lead to the inhibition of bone formation and the enhancement of bone resorption [4,5,6,7,8]. Increased oxidative stress due to estrogen deficiency may be a primary cause of the development of postmenopausal osteoporosis [7,8]. Antioxidant compounds, including many polyphenolic substances of natural origin that are commonly found in plant-based products, may help inhibit the progression of osteoporosis resulting from sex hormone deficiency as well as other conditions associated with increased oxidative stress [9,10]. Natural compounds with proven antioxidant properties include, among others, phenolic acids, an important group of bioactive compounds found in plants. Additionally, these compounds have been reported to exhibit anti-inflammatory effects, modulate glycemic control and enhance insulin sensitivity, improve cholesterol levels, reduce blood pressure, and offer protection against heart disease. Additionally, they have the potential to protect brain cells from damage, potentially slowing cognitive decline. Furthermore, certain phenolic acids have demonstrated promise in inhibiting cancer cell growth and preventing tumor development [11,12].

Experimental studies have demonstrated the beneficial effects of certain phenolic acids on the development of osteoporosis induced by estrogen deficiency [13,14,15,16]. The beneficial effect of a diet rich in phenolic acids on the skeletal system has also been observed in young rats with normal levels of sex hormones [17]. It has also been observed that some phenolic acids, such as caffeic acid, sinapic acid, and rosmarinic acid, may also induce increases in estrogen levels [18,19,20,21].

Rosmarinic acid and sinapic acid are examples of natural phenolic acids and are derivatives of hydroxycinnamic acid [22]. Rosmarinic acid is an ester of caffeic acid and 3,4-dihydroxyphenyl lactic acid, originally isolated from *Rosmarinus officinalis* L. (rosemary), and occurs primarily in plants of the Lamiaceae family, which are widely used as spices and medicinal plants (e.g., *Rosmarinus officinalis* L., *Melissa officinalis* L., *Mentha × piperita* L., *Salvia officinalis* L., *Thymus vulgaris* L.) [23]. Sinapic acid is commonly found in various edible plants (e.g., wheat, rice, spices, mustard seeds, citrus fruits, berries) [24]. Some plant materials containing rosmarinic acid or sinapic acid have been used in traditional medicine [25,26]. Limited literature data suggest a potential beneficial effect of rosmarinic acid and sinapic acid on the skeletal system. In vitro studies have shown that both rosmarinic acid [27,28] and sinapic acid [29,30] may stimulate osteogenesis. It has also been confirmed that rosmarinic acid may inhibit the differentiation of osteoclasts [28,31]. In in vivo studies, it has been demonstrated that rosmarinic acid improved bone mass in mice with soluble RANKL-induced bone loss [28], while sinapic acid increased bone regeneration in male rats with normal levels of sex hormones [29]. It should be noted that not all studies have shown beneficial effects of phenolic acids on the skeletal system. We have previously reported that caffeic acid worsened the mechanical properties of bones [15,32].

The effects of rosmarinic acid and sinapic acid on the skeletal system under conditions of estrogen deficiency have not been studied to date. In our previous studies, we demonstrated that both rosmarinic acid and sinapic acid, in addition to increasing estradiol levels, also improved oxidative stress parameters that were disrupted due to estrogen deficiency [19,20]. The aim of the study was to investigate the effects of rosmarinic acid and sinapic acid administered at doses achievable in a human diet and higher (pharmacological ones) on the skeletal system of rats with estrogen deficiency induced by bilateral ovariectomy, a commonly used animal model of postmenopausal osteoporosis.

## 2. Materials and Methods

### 2.1. Animals and Experimental Design

The experiments were performed on three-month-old female Wistar rats. The research was conducted with the approval of the Local Ethics Committee in Katowice (permission numbers: 38/2015, 148/2015, and 66/2016). The animals were obtained from the Center for Experimental Medicine, Medical University of Silesia, Katowice, Poland. The animals were maintained in a controlled laboratory environment according to EU Directive 2010/63. Throughout the duration of the experiment, the rats had unrestricted access to drinking water and a standard laboratory diet (Labofeed B, Wytwórnia Pasz Morawski, Kcynia, Poland). Details of the in vivo experiment were already reported [19,20].

The animals were divided into 7 groups (*n* = 10):

I—control rats that underwent a sham operation (SHAM);
II—control rats that underwent bilateral ovariectomy (OVX);
III—positive control rats: bilaterally ovariectomized, administered with estradiol at a dose of 0.2 mg/kg/day p.o. (OVX+E);
IV—bilaterally ovariectomized rats administered with rosmarinic acid at a dose of 10 mg/kg/day p.o. (OVX+RA10);
V—bilaterally ovariectomized rats administered with rosmarinic acid at a dose of 50 mg/kg/day p.o. (OVX+RA50);
VI—bilaterally ovariectomized rats administered with sinapic acid at a dose of 5 mg/kg/day p.o. (OVX+SA5);
VII—bilaterally ovariectomized rats administered with sinapic acid at a dose of 25 mg/kg/day p.o. (OVX+SA25).

Both ovariectomy and sham surgery were performed one week before the start of the drug administration under general anesthesia induced by intraperitoneal (i.p.) injection of a mixture of ketamine (Ketamina 10%, Biowet Puławy, Puławy, Poland) and xylazine (Xylapan, Vetoquinol Biowet, Gorzów Wlkp., Poland).

The investigated compounds: rosmarinic acid (Sigma-Aldrich, St. Louis, MO, USA), sinapic acid (Sigma-Aldrich, St. Louis, MO, USA), and estradiol hemihydrate (Estrofem, tablets, 2 mg of estradiol, Novo Nordisk A/S, Bagsvard, Denmark) were administered orally (by a gastric tube) to the rats in the morning (8–10 a.m.). Rosmarinic acid was either dissolved (10 mg/kg) or suspended (50 mg/kg) in tap water. Sinapic acid and estradiol were suspended in tap water. To all solutions and suspensions, Tween 20 (Sigma-Aldrich, St. Louis, MO, USA) was added (up to 1 µL/1 mL tap water). Control rats were administered tap water with the same quantity of Tween 20, at the same volume of 2 mL/kg. The phenolic acids, estradiol, or water were administered once daily for 28 days. The day after the last drug or vehicle administration, the rats, after overnight fasting, were sacrificed by cardiac exsanguination under general anesthesia induced by ketamine and xylazine, between 9 a.m. and 1 p.m. The bones (left and right femurs, left tibia, and L4 vertebra) were isolated and kept at a temperature below −20 °C. The blood serum was stored at −80 °C.

### 2.2. Bone Macrometric Parameters, Mass, Composition, and Mineralization Studies

In the isolated long bones, the macrometric parameters—length and diameter in the mid-length—were measured with the use of a digital caliper (Topex, Warsaw, Poland). The left bones and L4 vertebrae were weighed with an Adventurer Pro AV264CM analytical balance (Ohaus Europe GmbH, Greifensee, Switzerland).

To determine bone composition and mineralization, the left femur, left tibia, and L4 vertebra were lyophilized for ten days in FreeZone 6 lyophilizer (Labconco, Kansas City, MO, USA) at a pressure of 0.03 mbar and a temperature of −51 °C. Subsequently, the bones were mineralized (ashed) at 640 °C for 48 h in a L9/11/C6 muffle furnace (Nabertherm, Lilienthal, Germany). The lyophilized and mineralized bones were weighed using an Adventurer Pro AV264CM analytical balance (Ohaus Europe GmbH, Greifensee, Switzerland). Bone water mass was calculated by subtracting the bone mass after lyophilization from the bone mass. Mass of the organic bone substances was obtained by subtracting the mineral mass from the bone mass after lyophilization. The content of bone water, organic substances, and minerals was calculated as their ratio to bone mass.

The ashed bones were dissolved in 6 M HCl and subsequently diluted with deionized water for the determination of calcium, phosphorus, and magnesium content in the bone mineral. Measurements were conducted using an automatic biochemical analyzer (Mindray BS-240, Shenzhen, China) with commercially available kits: Pointe Scientific (Canton, MI, USA) for calcium and phosphorus, and Alpha Diagnostics Sp. z o.o. (Warsaw, Poland) for magnesium. The contents of calcium, phosphorus, and magnesium in the bone mineral were calculated.

Bone density was measured in the left tibia, left femur, and L4 vertebra using an Adventurer Pro AV264CM analytical balance equipped with a density determination kit (Ohaus Europe GmbH, Greifensee, Switzerland), based on Archimedes’ principle [33]. The bone mineral density was determined by calculating the ratio of bone mineral mass to bone volume.

### 2.3. Serum Biochemical Studies

Serum samples obtained from rats at the time of sacrifice were analyzed using ELISA tests for osteocalcin, a marker of bone formation (Rat-MID Osteocalcin EIA, Immunodiagnostic Systems Ltd., Boldon, Tyne and Wear, UK), and C-terminal telopeptide fragments of type I collagen, which are released during bone resorption (RatLaps EIA, Immunodiagnostic Systems Ltd., Boldon, Tyne and Wear, UK) in accordance with the manufacturer’s protocol.

Blood samples were also used to analyze the concentrations of sex hormones, selected biochemical parameters, and oxidative stress markers, and the results were reported in our previous publications [19,20].

### 2.4. Bone Mechanical Properties Studies

The mechanical properties of bone were assessed using an Instron 3342 500N apparatus (Instron, Norwood, MA, USA), with data analysis conducted by Bluehill 2 software version 2.14 (Instron, Norwood, MA, USA). Mechanical properties of the proximal metaphysis of the left tibia and diaphysis of the left femur were evaluated using three-point bending tests, as previously described [34,35,36], and mechanical properties of the femoral neck were assayed in a compression test [35].

Before the measurements, the proximal epiphysis was excised from the tibia. The load was applied perpendicular to the long axis of the bone, 3 mm from the proximal tibial metaphysis edge. The bone was supported at two points: the proximal metaphysis edge and the distal tibial diaphysis at the site of synostosis with the fibula. The distance between these two points was measured using a digital caliper (Topex, Warsaw, Poland) prior to the test. The test was initiated after applying a preload of 1 N, with a displacement rate of 0.01 mm/s and a sampling frequency of 100 Hz. The extrinsic parameters measured, which are dependent on the bone’s size, included load, displacement, and energy at the yield point (0.05% offset), as well as the maximum load point and fracture point. Additionally, the intrinsic parameters, which are independent of bone dimensions, were evaluated: stress at the yield point, maximum load point and fracture point, and Young’s modulus. To calculate the moment of inertia at the fracture site, the mean diameter of the tibial metaphysis was measured using a digital caliper (Topex), assuming the tibial metaphysis to be a circular beam.

To evaluate the mechanical properties of the femoral diaphysis, the femur was positioned on two support points with a distance of 16 mm between them. The load was applied perpendicularly to the bone’s long axis at the center of the femoral length. After pre-conditioning to ensure stable bone positioning (five cycles: load from 0 to 4 N, displacement rate of 0.01 mm/s), the mechanical test was performed (displacement rate of 0.01 mm/s, sampling rate of 100 Hz). The same parameters as for the tibia were measured. To calculate the moment of inertia at the fracture site, it was assumed that the femoral diaphysis was an elliptical pipe [37]. Transverse cross-section of the right femoral diaphysis was made at the femoral midpoint, and the internal and external diameters were measured using the OsteoMeasure system, which incorporated a microscope (Axio Imager.A1, Carl Zeiss, Göttingen, Germany), camera (DP71, Olympus, Tokyo, Japan), graphic tablet (Cintiq 22HD, Wacom, Kazo, Japan) and the software OsteoMeasure XP v1.3.0.1 (OsteoMetrics, Decatur, GA, USA).

The mechanical properties of the femoral neck were assayed in a compression test [34]. After the proximal part of the right femur (previously cut at mid-length) was fixed to a polymethyl methacrylate plate, the mechanical testing commenced (displacement rate of 0.01 mm/s, sampling rate of 100 Hz). The load was applied to the femoral head along the bone’s long axis, following a preload of 1 N, with a displacement rate of 0.01 mm/s. The maximum load, which induced the fracture of the femoral neck, was recorded.

### 2.5. Bone Histomorphometric Studies

Histomorphometric measurements were performed using the OsteoMeasure system described above. The histomorphometric parameters were presented according to the American Society for Bone and Mineral Research (ASBMR)’s standardized nomenclature [38].

The measurements of the transverse cross-sections of the femoral diaphysis (compact bone) were carried out on undecalcified, unstained preparations of the femoral diaphysis, prepared as previously described [35]. The transverse cross-sectional area of the marrow cavity (Ma.Ar), transverse cross-sectional area of the cortical bone (Ct.Ar), transverse cross-sectional area of the total diaphysis (Tt.Ar), and the Ma.Ar/Tt.Ar ratio were determined.

The measurements of the longitudinal cross-sections of the femoral epiphysis and metaphysis (cancellous bone) were performed on decalcified preparations stained with hematoxylin and eosin [39]. Bone volume/tissue volume ratio (BV/TV), trabecular thickness (Tb.Th), trabecular number (Tb.N), and trabecular separation (Tb.Sp) were evaluated.

### 2.6. Statistical Analysis

Statistical evaluations were conducted using the Statistica 13.3 program (Tibco Software Inc., Palo Alto, CA, USA). The obtained data were presented as mean ± standard deviation (SD). Statistical analysis was carried out with the use of the non-parametric Kruskal–Wallis test followed by the Mann–Whitney U test, because not all data met the assumptions of homogeneity of variance (Levene’s test) or normality (Shapiro–Wilk’s test) required for ANOVA. The results from all groups of ovariectomized rats were compared with those of the sham-operated control rats, and the results from rats treated with the phenolic acids or estradiol were compared with those of the ovariectomized controls. Results with *p* values ≤ 0.05 were considered to be statistically significant. Moreover, the results, which differed from the results of the control rats at *p* ≤ 0.1 in the Mann–Whitney U test (if the Kruskal–Wallis test resulted in *p* ≤ 0.1), were described as tendencies in the text.

## 3. Results

### 3.1. Effect of Rosmarinic Acid and Sinapic Acid on the Bone Macrometric Parameters, Mass, Composition, and Mineralization

As previously reported [19,20], the body mass gain in ovariectomized rats was greater than that in the sham-operated control rats. Consistently, the femoral bone length was significantly greater in the ovariectomized control rats and in ovariectomized rats treated with the phenolic acids (Table 1). Administration of estradiol (0.2 mg/kg) counteracted the increase in the femur length induced by estrogen deficiency.

Estrogen deficiency induced significant decreases in the bone density and bone mineral density in the femur, and in the bone density in the tibia (Table 2) and vertebra (Supplementary Materials, Table S1), in relation to the sham-operated control rats. Those effects were significantly counteracted by estradiol. Rosmarinic acid (10 mg/kg and 50 mg/kg) and sinapic acid (5 mg/kg and 25 mg/kg) did not affect those parameters in the femur and vertebra of ovariectomized rats. However, in the tibia, slight increases in bone density after the administration of sinapic acid at the lower dose (statistically significant), and rosmarinic acid at the higher dose (a tendency, *p* = 0.07), in relation to the ovariectomized controls were observed.

In the ovariectomized control rats, no significant changes in the bone mass and composition in relation to the sham-operated controls were demonstrated (only tendencies to decrease bone mineral content and to increase water content in the femur were noted). Only administration of estradiol tended (*p* = 0.06) to counteract the decrease in the mineral content in the femur of ovariectomized rats (Table 1).

### 3.2. Effect of Rosmarinic Acid and Sinapic Acid on Bone Turnover Markers

**Figure 1:**
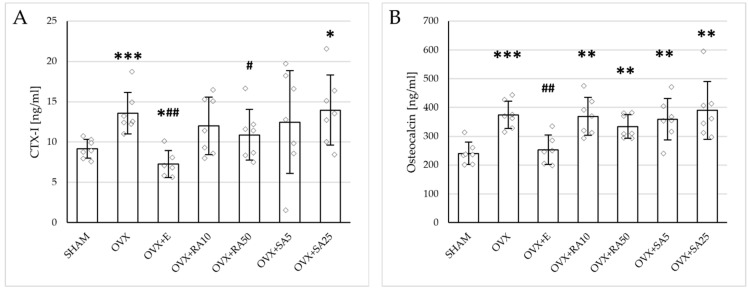
Effects of rosmarinic acid and sinapic acid administered orally for four weeks on the serum bone turnover marker concentration in rats with estrogen deficiency. (**A**) CTX-I (C-terminal telopeptide fragments of type I collagen). (**B**) Osteocalcin. The results are presented as means ± standard deviation (SD; *n* = 6–7). SHAM—control rats which underwent sham operation; OVX—control rats which underwent bilateral ovariectomy; OVX+E—bilaterally ovariectomized rats administered with estradiol at a dose of 0.2 mg/kg; OVX+RA10—bilaterally ovariectomized rats administered with rosmarinic acid at a dose of 10 mg/kg; OVX+RA50—bilaterally ovariectomized rats administered with rosmarinic acid at a dose of 50 mg/kg; OVX+SA5—bilaterally ovariectomized rats administered with sinapic acid at a dose of 5 mg/kg; OVX+SA25—bilaterally ovariectomized rats administered with sinapic acid at a dose of 25 mg/kg. Diamonds represent individual results. The Kruskal–Wallis test followed by the Mann–Whitney U test was used for statistical evaluation of the significance of the results. * *p* ≤ 0.05, ** *p* < 0.01, *** *p* < 0.001—in comparison with the SHAM control rats (SHAM group). # *p* ≤ 0.05, ## *p* < 0.01 in comparison with the OVX control rats (OVX group).

Significant increases in both the serum bone resorption (CTX-I) and formation (osteocalcin) markers were induced by estrogen deficiency; those effects were fully normalized by administration of estradiol (Figure 1). Rosmarinic acid at a lower dose and sinapic acid at both doses did not affect the concentration of CTX-I, whereas rosmarinic acid at a dose of 50 mg/kg slightly but significantly reduced its level in relation to the ovariectomized control rats. No significant changes in the osteocalcin concentration were observed following the treatment with either phenolic acid at any of the doses tested.

### 3.3. Effect of Rosmarinic Acid and Sinapic Acid on Mechanical Properties of Cancellous Bone (The Proximal Tibial Metaphysis)

**Figure 2:**
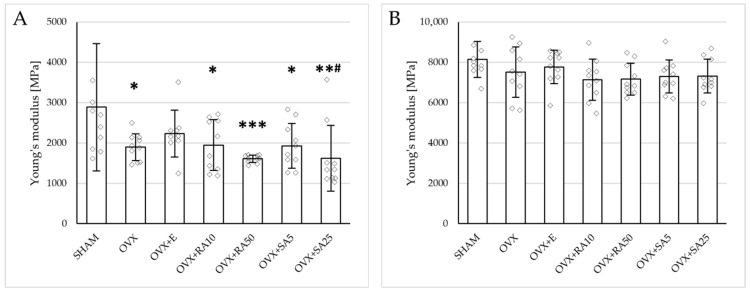
Effects of rosmarinic acid and sinapic acid administered orally for four weeks on the values of Young’s modulus in the proximal tibial metaphysis (**A**) and femoral diaphysis (**B**) in rats with estrogen deficiency. The results are presented as means ± standard deviation (SD; *n* = 9–10). SHAM—control rats which underwent sham operation; OVX—control rats which underwent bilateral ovariectomy; OVX+E—bilaterally ovariectomized rats administered with estradiol at a dose of 0.2 mg/kg; OVX+RA10—bilaterally ovariectomized rats administered with rosmarinic acid at a dose of 10 mg/kg; OVX+RA50—bilaterally ovariectomized rats administered with rosmarinic acid at a dose of 50 mg/kg; OVX+SA5—bilaterally ovariectomized rats administered with sinapic acid at a dose of 5 mg/kg; OVX+SA25—bilaterally ovariectomized rats administered with sinapic acid at a dose of 25 mg/kg. Diamonds represent individual results; some upper outliers (out of scale) are not shown. The Kruskal–Wallis test followed by the Mann–Whitney U test was used for statistical evaluation of the significance of the results. * *p* ≤ 0.05, ** *p* < 0.01, *** *p* < 0.001—in comparison with the SHAM control rats (SHAM group). # *p* ≤ 0.05 in comparison with the OVX control rats (OVX group).

**Figure 3:**
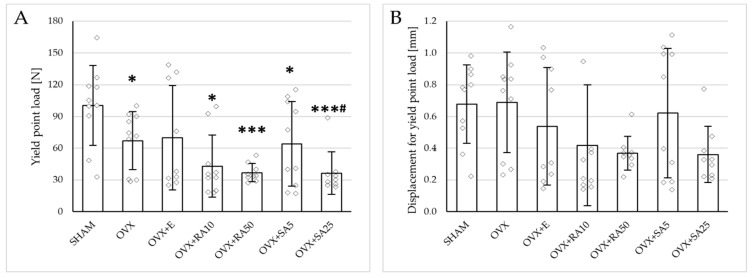

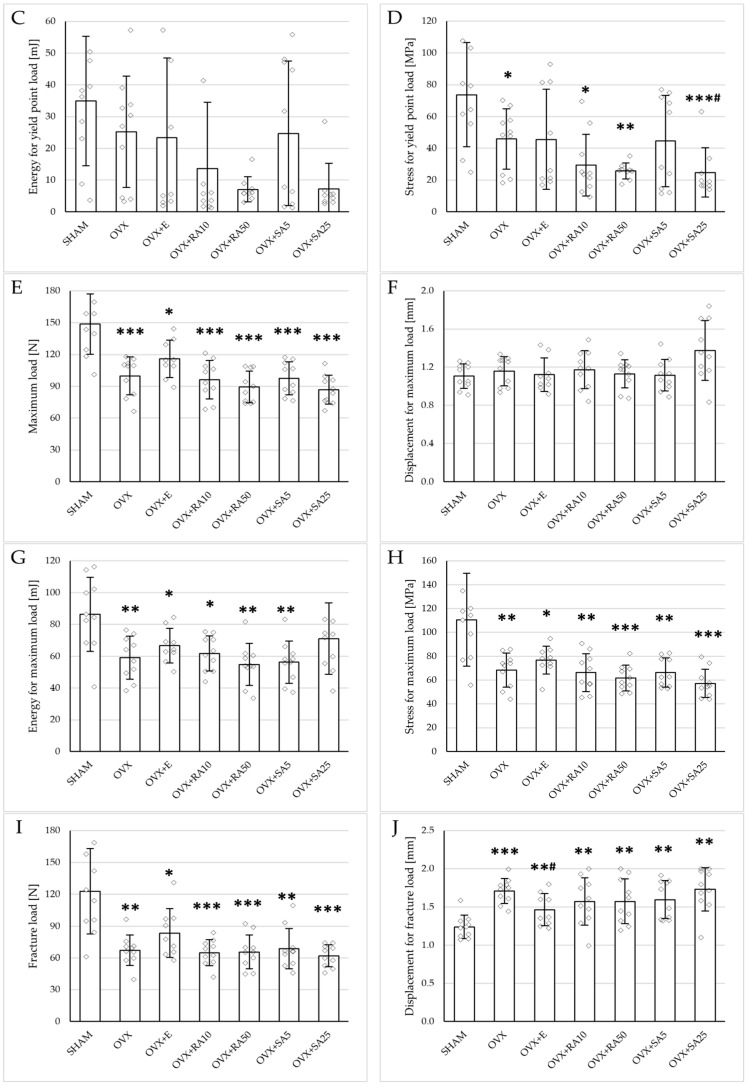

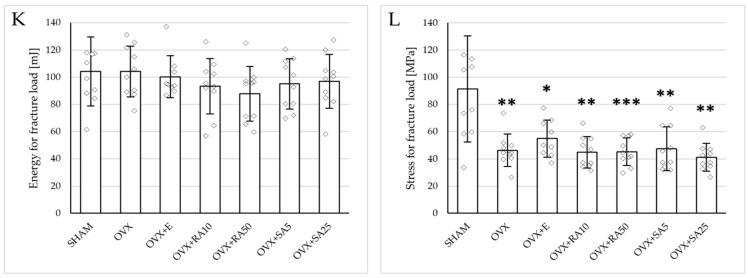
Effects of rosmarinic acid and sinapic acid administered orally for four weeks on the mechanical properties of the proximal tibial metaphysis in rats with estrogen deficiency. (**A**–**D**) Yield point load, displacement for yield point, energy for yield point, and stress for yield point. (**E**–**H**) Maximum load, displacement for maximum load, energy for maximum load, and stress for maximum load. (**I**–**L**) Fracture load, displacement for fracture load, energy for fracture load, and stress for fracture load. The results are presented as means ± standard deviation (SD; *n* = 9–10). SHAM—control rats which underwent sham operation; OVX—control rats which underwent bilateral ovariectomy; OVX+E—bilaterally ovariectomized rats administered with estradiol at a dose of 0.2 mg/kg; OVX+RA10—bilaterally ovariectomized rats administered with rosmarinic acid at a dose of 10 mg/kg; OVX+RA50—bilaterally ovariectomized rats administered with rosmarinic acid at a dose of 50 mg/kg; OVX+SA5—bilaterally ovariectomized rats administered with sinapic acid at a dose of 5 mg/kg; OVX+SA25—bilaterally ovariectomized rats administered with sinapic acid at a dose of 25 mg/kg. Diamonds represent individual results; some upper outliers (out of scale) are not shown. The Kruskal–Wallis test followed by the Mann–Whitney U test was used for statistical evaluation of the significance of the results. * *p* ≤ 0.05, ** *p* < 0.01, *** *p* < 0.001—in comparison with the SHAM control rats (SHAM group). # *p* ≤ 0.05 in comparison with the OVX control rats (OVX group).

Estrogen deficiency induced a profound worsening of the mechanical properties of the proximal tibial metaphysis, built mostly of cancellous bone, significantly decreasing Young’s modulus (Figure 2A), as well as the load and stress for the yield point, the maximum load point, and the fracture point in relation to the sham-operated control rats (Figure 3). Moreover, the energy for the maximum load point significantly decreased, and the displacement for the fracture point significantly increased. Administration of estradiol only slightly mitigated the changes in the mechanical properties induced by estrogen deficiency. A tendency toward an increase in Young’s modulus (*p* = 0.07) was observed compared to the ovariectomized control rats. A significant decrease in the displacement for the fracture point was also noted.

Administration of rosmarinic acid (10 mg/kg and 50 mg/kg) and sinapic acid (5 mg/kg and 25 mg/kg) did not counteract the effects of estrogen deficiency on the mechanical properties of cancellous bone. After the administration of the lower doses of both acids, there were no significant effects on the mechanical properties of cancellous bone in ovariectomized rats, whereas after the administration of the higher doses, the unfavorable effects of estrogen deficiency were intensified. Rosmarinic acid at a dose of 50 mg/kg showed tendencies to decrease the values of yield point load and stress (*p* = 0.07), energy for the yield point (*p* = 0.08), maximum load (*p* = 0.06), and Young’s modulus (*p* = 0.06) relative to the ovariectomized control rats. After the administration of sinapic acid at a dose of 25 mg/kg, the yield point load and stress, as well as Young’s modulus, significantly decreased in comparison with the ovariectomized control rats; there was also a tendency to reduce the energy for the yield point.

### 3.4. Effect of Rosmarinic Acid and Sinapic Acid on Mechanical Properties of Compact Bone (Femoral Diaphysis)

**Figure 4:**
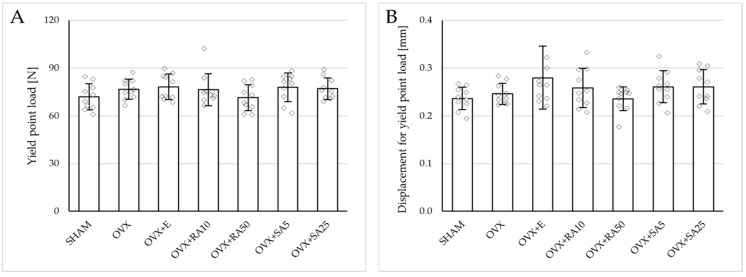

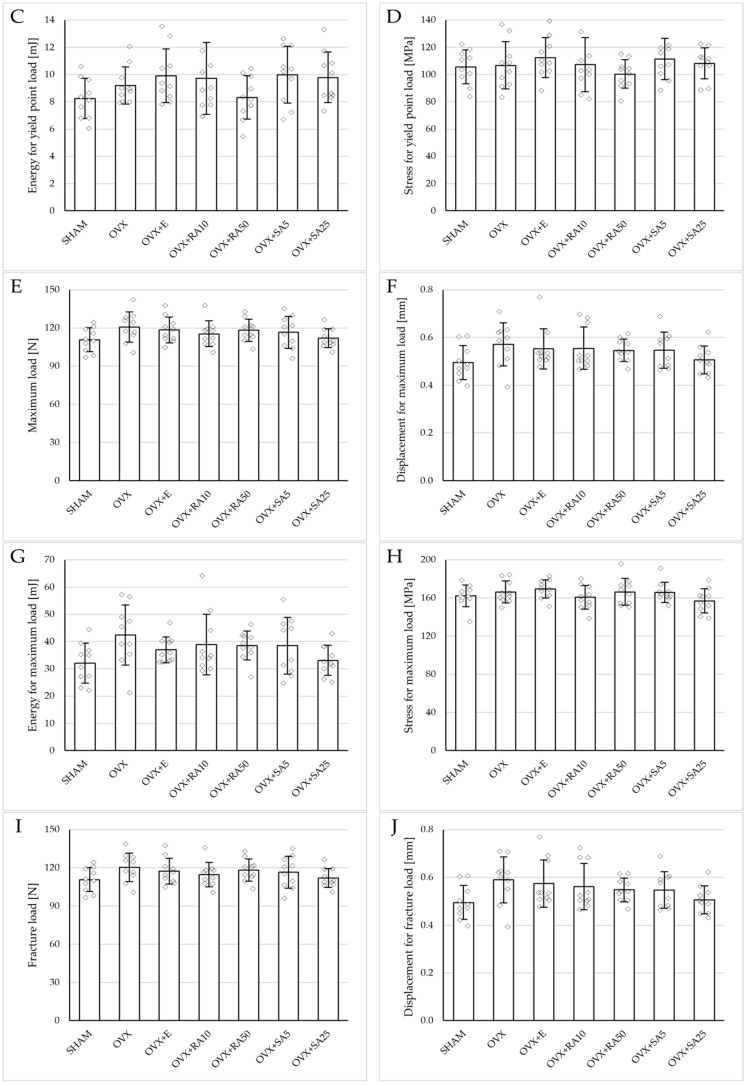

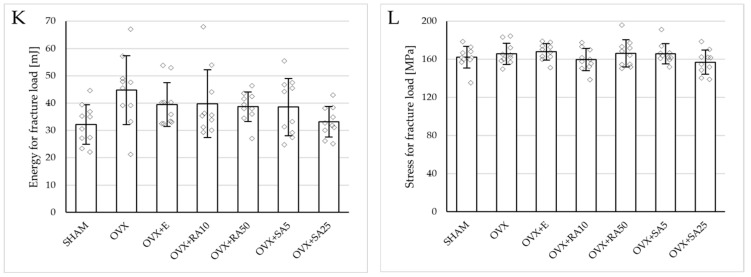
Effects of rosmarinic acid and sinapic acid administered orally for four weeks on the mechanical properties of the left femur in rats with estrogen deficiency. (**A**–**D**) Yield point load, displacement for yield point, energy for yield point, and stress for yield point. (**E**–**H**) Maximum load, displacement for maximum load, energy for maximum load, and stress for maximum load. (**I**–**L**) Fracture load, displacement for fracture load, energy for fracture load, and stress for fracture load. The results are presented as means ± standard deviation (SD; *n* = 10). SHAM—control rats which underwent sham operation; OVX—control rats which underwent bilateral ovariectomy; OVX+E—bilaterally ovariectomized rats administered with estradiol at a dose of 0.2 mg/kg; OVX+RA10—bilaterally ovariectomized rats administered with rosmarinic acid at a dose of 10 mg/kg; OVX+RA50—bilaterally ovariectomized rats administered with rosmarinic acid at a dose of 50 mg/kg; OVX+SA5—bilaterally ovariectomized rats administered with sinapic acid at a dose of 5 mg/kg; OVX+SA25—bilaterally ovariectomized rats administered with sinapic acid at a dose of 25 mg/kg. Diamonds represent individual results. The Kruskal–Wallis test followed by the Mann–Whitney U test was used for statistical evaluation of the significance of the results.

Estrogen deficiency did not significantly affect the investigated mechanical parameters of the femoral diaphysis in relation to the sham-operated controls (Figure 2B and Figure 4). Only a tendency to increase the energy for fracture load was demonstrated (*p* = 0.06); this effect was fully counteracted by administration of sinapic acid at a dose of 25 mg/kg. Estrogen deficiency and its treatments had no effect on the mechanical properties of the femoral neck, which is built of compact and cancellous bone (Supplementary Materials, Table S2).

### 3.5. Effect of Rosmarinic Acid and Sinapic Acid on Histomorphometric Parameters of the Femur

No significant effect on bone histomorphometric parameters of compact bone of the femoral diaphysis was observed in the ovariectomized control rats in relation to the sham-operated controls (Table 3). Administration of estradiol or the phenolic acids at all doses did not affect those parameters in ovariectomized rats.

In the femoral metaphysis, the ovariectomized control rats had significantly decreased values of BV/TV in relation to the sham-operated control rats (Table 4). There was also a tendency for the value of Tb.Sp to increase (*p* = 0.07). Administration of estradiol or the investigated phenolic acids at all doses did not counteract the deleterious effect of estrogen deficiency on the histomorphometric parameters of cancellous bone. In fact, rosmarinic acid at a dose of 50 mg/kg further (significantly) decreased the value of BV/TV, and also showed a tendency (*p* = 0.07) to reduce Tb.Th in relation to the ovariectomized control rats. There were no statistically significant changes induced by estrogen deficiency and the treatments in the histomorphometric parameters of the femoral epiphysis.

## 4. Discussion

It is widely believed that lifestyle factors, including adherence to an appropriate diet, can reduce the risk of bone fractures [40]. While dietary recommendations typically emphasize adequate intake of vitamin D and calcium, other nutrients may also play an important role in maintaining healthy bone tissue [41]. Consequently, research has been conducted to investigate the effects of various dietary components that may have a beneficial impact on bone metabolism [42,43,44,45]. Phenolic acids, commonly found in plant-based dietary components, including rosmarinic acid and sinapic acid, exhibit strong antioxidant properties [24,46]. Additionally, experimental studies have shown that these compounds can increase serum estrogen (estradiol) levels [19,20,21], suggesting a potential role for rosmarinic acid and sinapic acid in the prevention of postmenopausal osteoporosis. Therefore, the present study investigated the effects of rosmarinic acid and sinapic acid on the development of estrogen deficiency-induced osteoporosis in ovariectomized rats, a widely used model of postmenopausal osteoporosis [47].

In the present study, estrogen deficiency led to the development of osteoporotic changes in ovariectomized rats. Consistent with numerous previous studies [15,35,48,49,50,51], an increase in bone turnover was observed, as evidenced by elevated markers of both bone resorption and bone formation. Estrogen deficiency leads to an overall increase in bone remodeling activity, with increased rates of both bone resorption and formation. The dominant process is the intensification of bone resorption, which can be attributed to estrogen’s multifaceted regulatory functions: although it inhibits the differentiation of both osteoclasts and osteoblasts from their progenitor cells, it simultaneously induces apoptosis in osteoclasts and prevents apoptosis in osteoblasts and osteocytes [7]. The predominance of bone resorption in estrogen-deficient states is thought to arise not only from the absence of estrogen’s direct effects on osteoclasts but also from the loss of its immunomodulatory influence. This loss promotes a proinflammatory environment, characterized by increased production of cytokines that drive osteoclastogenesis, most notably receptor activator of nuclear factor kappa-B ligand (RANKL), tumor necrosis factor-alpha (TNF-α), interleukin-1 (IL-1), and interleukin-6 (IL-6) [52]. As a result of bone resorption exceeding bone formation, a reduction in bone density was observed, along with a marked deterioration in the mechanical properties of trabecular bone (the proximal tibial metaphysis). In contrast, no significant changes were found in the mechanical properties of cortical bone (the femoral diaphysis). The histomorphometric findings were consistent with the mechanical test results, showing deterioration of the microarchitecture in the femoral metaphysis, while no alterations were observed in cortical bone structure. Supplementation with estradiol (0.2 mg/kg) only slightly counteracted the changes induced by estrogen deficiency; although it significantly counteracted the effect on bone turnover markers and bone density, its influence on cancellous bone mechanical properties was marginal.

To counteract the development of osteoporosis, rosmarinic acid was administered at doses of 10 and 50 mg/kg, and sinapic acid at doses of 5 and 25 mg/kg, for a period of 4 weeks in the present study. The lower doses of rosmarinic acid and sinapic acid were within the range achievable through a human diet. The doses of rosmarinic acid (10 mg/kg) and sinapic acid (5 mg/kg) used in the present study in rats correspond to the doses of 1.6 mg/kg of rosmarinic acid and 0.8 mg/kg of sinapic acid in humans, taking into account the factor of 0.162 resulting from interspecies differences [53]. Rosmarinic acid is present in amounts of about 0.5–3% in commonly used herbs such as lemon balm, peppermint, and rosemary (consumed as infusions or spices) [54,55]. On the other hand, some vegetables and fruits are rich in sinapic acid, e.g., tronchuda cabbage contains about 180 µg/g [56] and strawberries contain about 445 µg/g [24]. It is therefore possible that an adult human weighing 70 kg consumes 5–10 g of herbs rich in rosmarinic acid and/or 150–250 g of vegetables or fruits rich in sinapic acid daily. The 5-fold higher doses used in the present study, i.e., 50 mg/kg of rosmarinic acid and 25 mg/g of sinapic acid, are pharmacological doses, in a range of doses often used in various experimental models in rats [57,58,59,60,61,62,63,64,65]. It should be noted that in readily available dietary supplements, various polyphenolic compounds are used, mainly in self-medication, in doses significantly exceeding the doses achievable in a regular diet [66,67]. The selected treatment duration was adequate to reveal the effects of various plant-derived compounds on the skeletal system of rats [35,39,68]. Considering the lifespan differences between species, a 4-week treatment period in rats was approximately equivalent to 2.5 years in humans [69].

Results of the present study indicated a lack of potential for rosmarinic and sinapic acids to counteract estrogen deficiency-induced bone damage. Only some slight beneficials effects of the administration of the phenolic acids on the skeletal system in conditions of estrogen deficiency were noted: at higher doses, sinapic acid tended to decrease the value of energy accumulated by femoral diaphysis to the fracture point (increased due to estrogen deficiency), and rosmarinic acid slightly but significantly decreased the serum level of a bone resorption marker (CTX-I). Moreover, increases in bone density were noted in the tibia, but not in other bones (femur, vertebra), after the administration of sinapic acid at a dose of 5 mg/kg (statistically significant) and rosmarinic acid at a dose of 50 mg/kg (a tendency; *p* = 0.07). Those results may be explained by the increase in the serum estradiol level. In fact, the inhibition of osteoclastogenesis in vitro was reported previously for rosmarinic acid [28,31]. To date, comparable findings have not been reported for sinapic acid, which did not exert an antiresorptive effect in the present study.

However, both investigated phenolic acids, administered at higher doses, unfavorably affected the bone mechanical properties of ovariectomized rats, further decreasing the values of Young’s modulus, yield point load, stress, energy and maximum load; the effects of sinapic acid were statistically significant, whereas rosmarinic acid strongly tended (*p* = 0.06–0.08) to worsen the bone mechanical parameters. Consistent with those results, rosmarinic acid (50 mg/kg) further worsened cancellous bone histomorphometric parameters, significantly decreasing BV/TV and tending to decrease Tb.Th (*p* = 0.07) in relation to the ovariectomized control rats.

To our knowledge, results indicating deleterious effects of the investigated phenolic acids on bone have not been reported so far. However, some unfavorable effects of other phenolic acids were demonstrated in our previous studies. In our very preliminary study [70], we observed a damaging effect of ferulic acid on the bones of ovariectomized rats. That was not confirmed in our further studies of the effect of ferulic acid and other phenolic acids [13]. We also reported in two experiments that caffeic acid worsened some bone mechanical properties of female rats with normal estrogen levels [15,32]; the effect was exerted by low doses (5 or 10 mg/kg) [15,32], but not by a high dose of the substance (50 mg/kg) [15].

Increases in the serum estradiol concentration demonstrated in rats whose bones were investigated in the present study [19,20] did not lead to an increase in bone strength. In contrast to estradiol, for which the serum concentration 24 h after the last administration was not significantly higher than in the control ovariectomized rats, the effect of the phenolic acids on the estradiol level lasted longer [19,20]. The lack of beneficial effect of rosmarinic acid and sinapic acid is consistent with the lack of effect of estradiol supplementation on bone mechanical properties, observed in the present study. It should be pointed out that both rosmarinic acid and sinapic acid improved the parameters related to oxidative stress, increasing the parameters of antioxidative defense and decreasing the parameters of oxidative damage in the serum (enhanced content of the reduced glutathione and decreased levels of advanced oxidation protein products, respectively) [19,20] and lenses (enhanced activity of glutathione reductase, and content of the reduced glutathione, as well as decreased levels of advanced oxidation protein products) [71]. Those data indicate that improvement in oxidative stress status did not counteract the deterioration of bone quality due to estrogen deficiency.

Our study does not explain the mechanism of the damaging effect exerted by higher doses of rosmarinic acid and sinapic acid on the skeletal system of estrogen-deficient rats. Taking into account two potential mechanisms by which they could hypothetically have been expected to beneficially affect bone, one may conclude that their effect on estrogen levels was too weak to counteract the effects of estrogen deficiency, and that the role of oxidative stress in the development of postmenopausal osteoporosis is of minor importance.

The most important limitation of the study is that it does not provide the mechanism of the demonstrated unfavorable effects of the phenolic acids at higher doses on the skeletal system, despite counteracting the increase in oxidative stress and increasing the serum estradiol levels. The phenolic acids were administered for 4 weeks; it is possible that a longer administration period would have provided more conclusive results. Another limitation is that the study concerned only female (ovariectomized) rats; the results may not be relevant to male organisms. Further studies are necessary.

The results of the present study suggest that supplementation with high doses of rosmarinic acid or sinapic acid may potentially have an adverse effect on the skeletal system in humans, particularly in postmenopausal women. Rosmarinic acid and sinapic acid, due to their reported health benefits, are recommended for use in order to counteract the negative effects of oxidative stress, which contributes to the aging process and the development of diseases [72,73,74]. Our findings indicate a need for extensive research on the safety issues of natural substances recommended for use as diet supplements based on experimental data.

## 5. Conclusions

In conclusion, the results of the present study demonstrated that administration of high doses of rosmarinic acid or sinapic acid slightly unfavorably affected the rat skeletal system in conditions of estrogen deficiency.

## Figures and Tables

**Table 1:** Effects of rosmarinic acid and sinapic acid administered orally for four weeks on femur length, diameter, mass, composition, mineralization, and density in rats with estrogen deficiency.

Parameter/Group	Sham- Operated Control Rats	Ovariectomized Control Rats	OVX+				
			E	RA10	RA50	SA5	SA25
Bone length (mm)	33.16 ± 0.49	34.15 ± 0.49 ***	33.50 ± 0.54 #	33.89 ± 0.58 *	33.82 ± 0.50 *	33.90 ± 0.80 *	33.39 ± 1.57 *
Bone diameter (mm)	3.31 ± 0.13	3.44 ± 0.12	3.31 ± 0.08	3.32 ± 0.12	3.36 ± 0.18	3.34 ± 0.09	3.34 ± 0.12
Bone mass (g)	0.671 ± 0.050	0.695 ± 0.048	0.681 ± 0.026	0.673 ± 0.065	0.673 ± 0.047	0.678 ± 0.048	0.670 ± 0.047
Bone mineral mass (g)	0.316 ± 0.021	0.314 ± 0.018	0.317 ± 0.013	0.308 ± 0.021	0.310 ± 0.019	0.311 ± 0.019	0.307 ± 0.018
Mass of bone mineral/bone mass ratio	0.471 ± 0.011	0.453 ± 0.015	0.465 ± 0.008	0.459 ± 0.015	0.462 ± 0.014	0.460 ± 0.008	0.458 ± 0.014
Mass of bone water/ bone mass ratio	0.298 ± 0.011	0.319 ± 0.017	0.307 ± 0.011	0.314 ± 0.017	0.308 ± 0.015	0.309 ± 0.009	0.312 ± 0.013
Mass of bone organic substances/bone mass ratio	0.231 ± 0.003	0.228 ± 0.007	0.228 ± 0.004	0.228 ± 0.005	0.230 ± 0.005	0.231 ± 0.005	0.229 ± 0.005
Calcium content (g/g of bone mineral)	0.405 ± 0.009	0.406 ± 0.014	0.407 ± 0.009	0.413 ± 0.020	0.408 ± 0.017	0.404 ± 0.008	0.409 ± 0.010
Phosphorus content (g/g of bone mineral)	0.157 ± 0.004	0.158 ± 0.005	0.157 ± 0.002	0.160 ± 0.006	0.159 ± 0.006	0.158 ± 0.004	0.159 ± 0.003
Magnesium content (g/g of bone mineral)	0.010 ± 0.003	0.012 ± 0.003	0.011 ± 0.003	0.010 ± 0.004	0.011 ± 0.004	0.010 ± 0.001	0.011 ± 0.002
Bone density (g/cm^3^)	1.629 ± 0.026	1.587 ± 0.026 **	1.618 ± 0.022 #	1.590 ± 0.031 *	1.600 ± 0.024 *	1.595 ± 0.023 **	1.597 ± 0.028 *
Bone mineral density (g/cm^3^)	0.767 ± 0.030	0.719 ± 0.035 **	0.752 ± 0.023 #	0.729 ± 0.036 *	0.739 ± 0.031 *	0.733 ± 0.022 *	0.732 ± 0.033 *

The results are presented as means ± standard deviation (SD; *n* = 10). OVX+E—bilaterally ovariectomized rats administered with estradiol at a dose of 0.2 mg/kg; OVX+RA10—bilaterally ovariectomized rats administered with rosmarinic acid at a dose of 10 mg/kg; OVX+RA50—bilaterally ovariectomized rats administered with rosmarinic acid at a dose of 50 mg/kg; OVX+SA5—bilaterally ovariectomized rats administered with sinapic acid at a dose of 5 mg/kg; OVX+SA25—bilaterally ovariectomized rats administered with sinapic acid at a dose of 25 mg/kg. The Kruskal–Wallis test followed by the Mann–Whitney U test was used for statistical evaluation of the significance of the results. * *p* ≤ 0.05, ** *p* < 0.01, *** *p* < 0.001—in comparison with the sham-operated control rats (SHAM group). # *p* ≤ 0.05 in comparison with the OVX control rats (OVX group).

**Table 2:** Effects of rosmarinic acid and sinapic acid administered orally for four weeks on tibia length, diameter, mass, composition, mineralization, and density in rats with estrogen deficiency.

Parameter/Group	Sham- Operated Control Rats	Ovariectomized Control Rats	OVX+				
			E	RA10	RA50	SA5	SA25
Bone length (mm)	36.99 ± 0.67	37.48 ± 1.30	37.54 ± 0.52	37.59 ± 0.94	37.53 ± 0.52	37.57 ± 0.73	37.71 ± 0.85
Bone diameter (mm)	2.74 ± 0.07	2.75 ± 0.11	2.70 ± 0.07	2.75 ± 0.10	2.76 ± 0.17	2.78 ± 0.08	2.79 ± 0.14
Bone mass (g)	0.522 ± 0.041	0.529 ± 0.047	0.521 ± 0.034	0.510 ± 0.036	0.513 ± 0.043	0.518 ± 0.034	0.513 ± 0.031
Bone mineral mass (g)	0.243 ± 0.017	0.238 ± 0.014	0.240 ± 0.012	0.236 ± 0.013	0.236 ± 0.015	0.239 ± 0.014	0.233 ± 0.013
Mass of bone mineral/ bone mass ratio	0.465 ± 0.016	0.452 ± 0.017	0.464 ± 0.016	0.463 ± 0.016	0.461 ± 0.018	0.462 ± 0.009	0.454 ± 0.017
Mass of bone water/ bone mass ratio	0.293 ± 0.014	0.305 ± 0.020	0.291 ± 0.019	0.295 ± 0.020	0.297 ± 0.016	0.296 ± 0.007	0.306 ± 0.014
Mass of bone organic substances/bone mass ratio	0.242 ± 0.006	0.243 ± 0.008	0.244 ± 0.006	0.242 ± 0.006	0.242 ± 0.006	0.242 ± 0.005	0.240 ± 0.006
Calcium content (g/g of bone mineral)	0.409 ± 0.013	0.404 ± 0.009	0.410 ± 0.009	0.413 ± 0.018	0.408 ± 0.024	0.397 ± 0.027	0.413 ± 0.010
Phosphorus content (g/g of bone mineral)	0.158 ± 0.004	0.158 ± 0.005	0.159 ± 0.003	0.159 ± 0.008	0.164 ± 0.024	0.154 ± 0.013	0.166 ± 0.015
Magnesium content (g/g of bone mineral)	0.008 ± 0.002	0.011 ± 0.002	0.011 ± 0.002	0.008 ± 0.003	0.010 ± 0.003	0.010 ± 0.002	0.009 ± 0.002
Bone density (g/cm^3^)	1.632 ± 0.023	1.580 ± 0.024 ***	1.609 ± 0.033 #	1.593 ± 0.027 **	1.604 ± 0.032 *	1.605 ± 0.021 *#	1.600 ± 0.037 *
Bone mineral density (g/cm^3^)	0.759 ± 0.035	0.729 ± 0.072	0.749 ± 0.037	0.737 ± 0.036	0.739 ± 0.040	0.740 ± 0.019	0.726 ± 0.040

The results are presented as means ± standard deviation (SD; *n* = 9–10). OVX+E—bilaterally ovariectomized rats administered with estradiol at a dose of 0.2 mg/kg; OVX+RA10—bilaterally ovariectomized rats administered with rosmarinic acid at a dose of 10 mg/kg; OVX+RA50—bilaterally ovariectomized rats administered with rosmarinic acid at a dose of 50 mg/kg; OVX+SA5—bilaterally ovariectomized rats administered with sinapic acid at a dose of 5 mg/kg; OVX+SA25—bilaterally ovariectomized rats administered with sinapic acid at a dose of 25 mg/kg. The Kruskal–Wallis test followed by the Mann–Whitney U test was used for statistical evaluation of the significance of the results. * *p* ≤ 0.05, ** *p* < 0.01, *** *p* < 0.001—in comparison with the sham-operated control rats (SHAM group). # *p* ≤ 0.05 in comparison with the OVX control rats (OVX group).

**Table 3:** Effects of rosmarinic acid and sinapic acid administered orally for four weeks on the histomorphometric parameters of compact bone in the femoral diaphysis in rats with estrogen deficiency.

Parameter/Group		Sham- Operated Control Rats	Ovariectomized Control Rats	OVX+				
				E	RA10	RA50	SA5	SA25
diaphysis	Ct.Ar (mm^2^)	4.879 ± 0.380	5.108 ± 0.283	5.025 ± 0.168	5.075 ± 0.275	5.013 ± 0.432	5.061 ± 0.352	5.070 ± 0.301
	Ma.Ar (mm^2^)	3.269 ± 0.340	3.489 ± 0.471	3.149 ± 0.348	3.282 ± 0.386	3.316 ± 0.415	3.291 ± 0.409	3.267 ± 0.413
	Tt.Ar (mm^2^)	8.149 ± 0.566	8.597 ± 0.675	8.175 ± 0.400	8.357 ± 0.591	8.330 ± 0.803	8.352 ± 0.592	8.337 ± 0.493
	Ma.Ar/Tt.Ar	0.401 ± 0.029	0.405 ± 0.026	0.384 ± 0.026	0.392 ± 0.022	0.397 ± 0.019	0.393 ± 0.032	0.391 ± 0.034

The results are presented as means ± standard deviation (SD; *n* = 10). OVX+E—bilaterally ovariectomized rats administered with estradiol at a dose of 0.2 mg/kg; OVX+RA10—bilaterally ovariectomized rats administered with rosmarinic acid at a dose of 10 mg/kg; OVX+RA50—bilaterally ovariectomized rats administered with rosmarinic acid at a dose of 50 mg/kg; OVX+SA5—bilaterally ovariectomized rats administered with sinapic acid at a dose of 5 mg/kg; OVX+SA25—bilaterally ovariectomized rats administered with sinapic acid at a dose of 25 mg/kg. Ct.Ar—transverse cross-sectional area of the cortical bone; Ma.Ar—transverse cross-sectional area of the marrow cavity; Ma.Ar/Tt.Ar—transverse cross-sectional area of the marrow cavity/total diaphysis area ratio. The Kruskal–Wallis test followed by the Mann–Whitney U test was used for statistical evaluation of the significance of the results.

**Table 4:** Effects of rosmarinic acid and sinapic acid administered orally for four weeks on the histomorphometric parameters of cancellous bone in the femoral metaphysis and epiphysis in rats with estrogen deficiency.

Parameter/Group		Sham-Operated Control Rats	Ovariectomized Control Rats	OVX+				
				E	RA10	RA50	SA5	SA25
metaphysis	BV/TV (%)	45.1 ± 4.0	38.4 ± 2.3 **	38.5 ± 2.3 **	37.0 ± 4.6 **	36.5 ± 3.8 **#	35.3 ± 3.9 ***	37.5 ± 4.9 **
	Tb.Th (μm)	52.8 ± 4.7	50.6 ± 8.1	48.5 ± 4.4	45.4 ± 7.3	43.5 ± 4.9	44.8 ± 6.8	47.4 ± 7.5
	Tb.Sp (μm)	64.5 ± 7.2	81.0 ± 12.4	77.5 ± 7.4	77.4 ± 10.0	75.8 ± 7.9	82.3 ± 12.1	79.0 ± 7.8
	Tb.N (1/mm)	8.56 ± 0.59	7.74 ± 1.09	7.99 ± 0.68	8.23 ± 0.90	8.43 ± 0.63	7.98 ± 1.03	7.95 ± 0.59
epiphysis	BV/TV (%)	31.6 ± 4.5	30.5 ± 3.1	33.0 ± 7.0	29.6 ± 5.0	29.7 ± 6.5	33.0 ± 8.7	30.2 ± 5.6
	Tb.Th (μm)	64.4 ± 8.1	62.7 ± 5.3	62.9 ± 14.5	60.8 ± 10.4	59.4 ± 15.5	64.9 ± 14.1	64.8 ± 15.2
	Tb.Sp (μm)	141.0 ± 21.5	143.6 ± 17.0	129.9 ± 32.9	146.0 ± 25.1	140.3 ± 21.0	136.8 ± 38.4	153.0 ± 46.2
	Tb.N (1/mm)	4.92 ± 0.58	4.88 ± 0.42	5.38 ± 1.22	4.92 ± 0.73	5.08 ± 0.60	5.14 ± 1.01	4.80 ± 0.99

The results are presented as means ± standard deviation (SD; *n* = 8–10). OVX+E—bilaterally ovariectomized rats administered with estradiol at a dose of 0.2 mg/kg; OVX+RA10—bilaterally ovariectomized rats administered with rosmarinic acid at a dose of 10 mg/kg; OVX+RA50—bilaterally ovariectomized rats administered with rosmarinic acid at a dose of 50 mg/kg; OVX+SA5—bilaterally ovariectomized rats administered with sinapic acid at a dose of 5 mg/kg; OVX+SA25—bilaterally ovariectomized rats administered with sinapic acid at a dose of 25 mg/kg. BV/TV—bone volume/tissue volume ratio; Tb.Th—trabecular thickness; Tb.Sp—trabecular separation; Tb.N—trabecular number. The Kruskal–Wallis test followed by the Mann–Whitney U test was used for statistical evaluation of the significance of the results. ** *p* < 0.01, *** *p* < 0.001—in comparison with the sham-operated control rats (SHAM group). # *p* ≤ 0.05 in comparison with the ovariectomized control rats.

## Data Availability

The original contributions presented in this study are included in the article. Further inquiries can be directed to the corresponding authors.

## Supplementary Materials

The following supporting information can be downloaded at: https://www.mdpi.com/article/10.3390/nu18020301/s1.

## Abbreviations

The following abbreviations are used in this manuscript:

BV/TV	Bone volume/tissue volume ratio
Ct.Ar	Transverse cross-sectional area of the cortical bone
CTX-I	C-terminal telopeptide fragments of type I collagen
i.p.	Intraperitoneal injection
IL-1	Interleukin-1
IL-6	Interleukin-6
Ma.Ar	Transverse cross-sectional area of the marrow cavity
Ma.Ar/Tt.Ar	Transverse cross-sectional area of the marrow cavity/total diaphysis area ratio
OVX	Rats that underwent bilateral ovariectomy
p.o.	*Per os* (orally)
RA	Rosmarinic acid
RANKL	Receptor activator of nuclear factor kappa-B ligand
SA	Sinapic acid
SHAM	Rats that underwent a sham operation
Tb.N	Trabecular number
Tb.Sp	Trabecular separation
Tb.Th	Trabecular thickness
TNF-α	Tumor necrosis factor-alpha

## References

[1] Sözen T., Özışık L., Başaran N.Ç. (2017). An Overview and Management of Osteoporosis. Eur. J. Rheumatol..

[2] Xiao P.L., Cui A.Y., Hsu C.J., Peng R., Jiang N., Xu X.H., Ma Y.G., Liu D., Lu H.D. (2022). Global, Regional Prevalence, and Risk Factors of Osteoporosis According to the World Health Organization Diagnostic Criteria: A Systematic Review and Meta-Analysis. Osteoporos. Int..

[3] Walker M., Shane E. (2023). Postmenopausal Osteoporosis. N. Engl. J. Med..

[4] Sobh M.M., Abdalbary M., Elnagar S., Nagy E., Elshabrawy N., Abdelsalam M., Asadipooya K., El-Husseini A. (2022). Secondary Osteoporosis and Metabolic Bone Diseases. J. Clin. Med..

[5] Azizieh F.Y., Shehab D., Jarallah K.A., Gupta R., Raghupathy R. (2019). Circulatory Levels of RANKL, OPG, and Oxidative Stress Markers in Postmenopausal Women with Normal or Low Bone Mineral Density. Biomark. Insights.

[6] Cervellati C., Bonaccorsi G., Cremonini E., Romani A., Fila E., Castaldini M.C., Ferrazzini S., Giganti M., Massari L. (2014). Oxidative Stress and Bone Resorption Interplay as a Possible Trigger for Postmenopausal Osteoporosis. Biomed Res. Int..

[7] Manolagas S.C. (2010). From Estrogen-Centric to Aging and Oxidative Stress: A Revised Perspective of the Pathogenesis of Osteoporosis. Endocr. Rev..

[8] Zhang C., Li H., Li J., Hu J., Yang K., Tao L. (2023). Oxidative Stress: A Common Pathological State in a High-Risk Population for Osteoporosis. Biomed. Pharmacother..

[9] Dudarić L., Fužinac-Smojver A., Muhvić D., Giacometti J. (2015). The Role of Polyphenols on Bone Metabolism in Osteoporosis. Food Res. Int..

[10] Sirše M. (2022). Effect of Dietary Polyphenols on Osteoarthritis—Molecular Mechanisms. Life.

[11] Kumar N., Goel N. (2019). Phenolic Acids: Natural Versatile Molecules with Promising Therapeutic Applications. Biotechnol. Reports.

[12] Oluwole O., Fernando W.B., Lumanlan J., Ademuyiwa O., Jayasena V. (2022). Role of Phenolic Acid, Tannins, Stilbenes, Lignans and Flavonoids in Human Health—A Review. Int. J. Food Sci. Technol..

[13] Folwarczna J., Zych M., Burczyk J., Trzeciak H., Trzeciak H.I. (2009). Effects of Natural Phenolic Acids on the Skeletal System of Ovariectomized Rats. Planta Med..

[14] Caviness P.C., Lazarenko O.P., Blackburn M.L., Chen J.F., Randolph C.E., Zabaleta J., Zhan F., Chen J.R. (2024). Phenolic Acids Prevent Sex-Steroid Deficiency-Induced Bone Loss and Bone Marrow Adipogenesis in Mice. J. Nutr. Biochem..

[15] Folwarczna J., Pytlik M., Zych M., Cegiela U., Nowinska B., Kaczmarczyk-Sedlak I., Śliwiński L., Trzeciak H., Trzeciak H.I. (2015). Effects of Caffeic and Chlorogenic Acids on the Rat Skeletal System. Eur. Rev. Med. Pharmacol. Sci..

[16] Yamaguchi M., Lai Y.L., Uchiyama S., Nakagawa T. (2008). Oral Administration of Phytocomponent P-Hydroxycinnamic Acid Prevents Bone Loss in Ovariectomized Rats. Mol. Cell. Biochem..

[17] Chen J.R., Lazarenko O.P., Wu X., Kang J., Blackburn M.L., Shankar K., Badger T.M., Ronis M.J. (2010). Dietary-Induced Serum Phenolic Acids Promote Bone Growth via P38 MAPK/β-Catenin Canonical Wnt Signaling. J. Bone Miner. Res..

[18] Zych M., Folwarczna J., Trzeciak H.I. (2009). Natural Phenolic Acids May Increase Serum Estradiol Level in Ovariectomized Rats. Acta Biochim. Pol..

[19] Zych M., Kaczmarczyk-Sedlak I., Wojnar W., Folwarczna J. (2018). The Effects of Sinapic Acid on the Development of Metabolic Disorders Induced by Estrogen Deficiency in Rats. Oxid. Med. Cell. Longev..

[20] Zych M., Kaczmarczyk-Sedlak I., Wojnar W., Folwarczna J. (2019). Effect of Rosmarinic Acid on the Serum Parameters of Glucose and Lipid Metabolism and Oxidative Stress in Estrogen-Deficient Rats. Nutrients.

[21] Ghasemzadeh A., Khaki A., Farzadi L., Khaki A., Marjani M., Ashteani H.A., Hamdi B.A., Ghadamkheir E., Naeimikararoudi M., Ouladsahebmadarek E. (2011). Effect of Rosmarinic Acid on Estrogen, FSH and LH in Female Diabetic Rats. Afr. J. Pharm. Pharmacol..

[22] Alam M.A., Subhan N., Hossain H., Hossain M., Reza H.M., Rahman M.M., Ullah M.O. (2016). Hydroxycinnamic Acid Derivatives: A Potential Class of Natural Compounds for the Management of Lipid Metabolism and Obesity. Nutr. Metab..

[23] Alagawany M., Abd El-Hack M.E., Farag M.R., Gopi M., Karthik K., Malik Y.S., Dhama K. (2017). Rosmarinic Acid: Modes of Action, Medicinal Values and Health Benefits. Anim. Health Res. Rev..

[24] Pandi A., Kalappan V.M. (2021). Pharmacological and Therapeutic Applications of Sinapic Acid—An Updated Review. Mol. Biol. Rep..

[25] Dang R., Guan H., Wang C. (2023). Sinapis Semen: A Review on Phytochemistry, Pharmacology, Toxicity, Analytical Methods and Pharmacokinetics. Front. Pharmacol..

[26] Ortiz-Mendoza N., Martínez-Gordillo M.J., Martínez-Ambriz E., Basurto-Peña F.A., González-Trujano M.E., Aguirre-Hernández E. (2023). Ethnobotanical, Phytochemical, and Pharmacological Properties of the Subfamily Nepetoideae (Lamiaceae) in Inflammatory Diseases. Plants.

[27] Jeong M.J., Lim D.S., Kim S.O., Park C., Choi Y.H., Jeong S.J. (2021). Effect of Rosmarinic Acid on Differentiation and Mineralization of MC3T3-E1 Osteoblastic Cells on Titanium Surface. Anim. Cells Syst..

[28] Lee J.-W., Asai M., Jeon S.-K., Iimura T., Yonezawa T., Cha B.-Y., Woo J.T., Yamaguchi A. (2015). Rosmarinic Acid Exerts an Antiosteoporotic Effect in the RANKL-induced Mouse Model of Bone Loss by Promotion of Osteoblastic Differentiation and Inhibition of Osteoclastic Differentiation. Mol. Nutr. Food Res..

[29] Balagangadharan K., Trivedi R., Vairamani M., Selvamurugan N. (2019). Sinapic Acid-Loaded Chitosan Nanoparticles in Polycaprolactone Electrospun Fibers for Bone Regeneration in Vitro and in Vivo. Carbohydr. Polym..

[30] Sadhasivam D.R., Soundararajan S., Elumalai S., Karuppiah P., Abdullah AL-Dhabi N. (2020). Prophylactic Supplementation of Sinapic Acid Ameliorates Zoledronic Acid Induced Changes in Osteoblast Survival and Differentiation. Biocatal. Agric. Biotechnol..

[31] Omori A., Yoshimura Y., Deyama Y., Suzuki K. (2015). Rosmarinic Acid and Arbutin Suppress Osteoclast Differentiation by Inhibiting Superoxide and NFATc1 Downregulation in RAW 264.7 Cells. Biomed. Rep..

[32] Zych M., Folwarczna J., Pytlik M., Śliwiński L., Gołden M.A., Burczyk J., Trzeciak H.I. (2010). Administration of Caffeic Acid Worsened Bone Mechanical Properties in Female Rats. Planta Med..

[33] Keenan M.J., Hegsted M., Jones K.L., Delany J.P., Kime J.C., Melancon L.E., Tulley R.T., Hong K.D. (1997). Comparison of Bone Density Measurement Techniques: DXA and Archimedes’ Principle. J. Bone Miner. Res..

[34] Stürmer E.K., Seidlová-Wuttke D., Sehmisch S., Rack T., Wille J., Frosch K.H., Wuttke W., Stürmer K.M. (2006). Standardized Bending and Breaking Test for the Normal and Osteoporotic Metaphyseal Tibias of the Rat: Effect of Estradiol, Testosterone, and Raloxifene. J. Bone Miner. Res..

[35] Folwarczna J., Pytlik M., Zych M., Cegiela U., Kaczmarczyk-Sedlak I., Nowińska B., Śliwiński L. (2013). Favorable Effect of Moderate Dose Caffeine on the Skeletal System in Ovariectomized Rats. Mol. Nutr. Food Res..

[36] Turner C.H., Burr D.B. (1993). Basic Biomechanical Measurements of Bone: A Tutorial. Bone.

[37] Kiebzak G.M., Smith R., Gundberg C.C., Howe J.C., Sacktor B. (1988). Bone Status of Senescent Male Rats: Chemical, Morphometric, and Mechanical Analysis. J. Bone Miner. Res..

[38] Dempster D.W., Compston J.E., Drezner M.K., Glorieux F.H., Kanis J.A., Malluche H., Meunier P.J., Ott S., Recker R.R., Parfitt M. (2013). Standardized nomenclature, symbols, and units for bone histomorphometry: A 2012 update of the report of the ASBMR Histomorphometry Nomenclature Committee. J. Bone Miner. Res..

[39] Londzin P., Siudak S., Cegieła U., Pytlik M., Janas A., Waligóra A., Folwarczna J. (2018). Phloridzin, an Apple Polyphenol, Exerted Unfavorable Effects on Bone and Muscle in an Experimental Model of Type 2 Diabetes in Rats. Nutrients.

[40] Wilson-Barnes S.L., Lanham-New S.A., Lambert H. (2022). Modifiable Risk Factors for Bone Health & Fragility Fractures. Best Pract. Res. Clin. Rheumatol..

[41] Liu X., Wu Y., Bennett S., Zou J., Xu J., Zhang L. (2024). The Effects of Different Dietary Patterns on Bone Health. Nutrients.

[42] Nicolin V., De Tommasi N., Nori S.L., Costantinides F., Berton F., Di Lenarda R. (2019). Modulatory Effects of Plant Polyphenols on Bone Remodeling: A Prospective View from the Bench to Bedside. Front. Endocrinol..

[43] Xu Q., Cao Z., Xu J.Q., Dai M., Zhang B., Lai Q., Liu X. (2022). Effects and Mechanisms of Natural Plant Active Compounds for the Treatment of Osteoclast-Mediated Bone Destructive Diseases. J. Drug Target..

[44] Lin B., Xu P., Zheng J., Deng X., Ye Q., Huang Z., Wang N. (2022). Effects and Mechanisms of Natural Alkaloids for Prevention and Treatment of Osteoporosis. Front. Pharmacol..

[45] Hanga-Farcaș A., Miere Groza F., Filip G.A., Clichici S., Fritea L., Vicaș L.G., Marian E., Pallag A., Jurca T., Filip S.M. (2023). Phytochemical Compounds Involved in the Bone Regeneration Process and Their Innovative Administration: A Systematic Review. Plants.

[46] Noor S., Mohammad T., Rub M.A., Raza A., Azum N., Yadav D.K., Hassan M.I., Asiri A.M. (2022). Biomedical Features and Therapeutic Potential of Rosmarinic Acid. Arch. Pharm. Res..

[47] Lelovas P.P., Xanthos T.T., Thorma S.E., Lyritis G.P., Dontas I.A. (2008). The Laboratory Rat as an Animal Model for Osteoporosis Research. Comp. Med..

[48] Abo-Elenin M.H.H., Kamel R., Nofal S., Ahmed A.A.E. (2025). The Crucial Role of Beta-Catenin in the Osteoprotective Effect of Semaglutide in an Ovariectomized Rat Model of Osteoporosis. Naunyn. Schmiedebergs. Arch. Pharmacol..

[49] Folwarczna J., Zych M., Nowińska B., Pytlik M., Bialik M., Jagusiak A., Lipecka-Karcz M., Matysiak M. (2016). Effect of Diosgenin, a Steroidal Sapogenin, on the Rat Skeletal System. Acta Biochim. Pol..

[50] Puzio I., Graboś D., Bieńko M., Radzki R.P., Nowakiewicz A., Kosior-Korzecka U. (2021). Camelina Oil Supplementation Improves Bone Parameters in Ovariectomized Rats. Animals.

[51] Hossain M., Sultana T., Moon J.E., Moon G.S., Jeong J.H. (2025). Anti-Osteoporotic Potential of a Probiotic Mixture Containing Limosilactobacillus Reuteri and Weissella Cibaria in Ovariectomized Rats. Sci. Rep..

[52] Uehara I.A., Soldi L.R., Silva M.J.B. (2020). Current Perspectives of Osteoclastogenesis through Estrogen Modulated Immune Cell Cytokines. Life Sci..

[53] Nair A., Morsy M.A., Jacob S. (2018). Dose Translation between Laboratory Animals and Human in Preclinical and Clinical Phases of Drug Development. Drug Dev. Res..

[54] Fecka I., Turek S. (2007). Determination of Water-Soluble Polyphenolic Compounds in Commercial Herbal Teas from Lamiaceae: Peppermint, Melissa, and Sage. J. Agric Food Chem..

[55] Wang H., Provan G.J., Helliwell K. (2004). Determination of Rosmarinic Acid and Caffeic Acid in Aromatic Herbs by HPLC. Food Chem..

[56] Nićiforović N., Abramovič H. (2014). Sinapic Acid and Its Derivatives: Natural Sources and Bioactivity. Compr. Rev. Food Sci. Food Saf..

[57] Rocha J., Eduardo-Figueira M., Barateiro A., Fernandes A., Brites D., Bronze R., Duarte C.M., Serra A.T., Pinto R., Freitas M. (2015). Anti-Inflammatory Effect of Rosmarinic Acid and an Extract of Rosmarinus Officinalis in Rat Models of Local and Systemic Inflammation. Basic Clin. Pharmacol. Toxicol..

[58] Silambarasan T., Manivannan J., Priya M.K., Suganya N., Chatterjee S., Raja B. (2014). Sinapic Acid Prevents Hypertension and Cardiovascular Remodeling in Pharmacological Model of Nitric Oxide Inhibited Rats. PLoS ONE.

[59] Govindaraj J., Sorimuthu Pillai S. (2015). Rosmarinic Acid Modulates the Antioxidant Status and Protects Pancreatic Tissues from Glucolipotoxicity Mediated Oxidative Stress in High-Fat Diet: Streptozotocin-Induced Diabetic Rats. Mol. Cell. Biochem..

[60] Zhang T., Liu C., Ma S., Gao Y., Wang R. (2020). Protective Effect and Mechanism of Action of Rosmarinic Acid on Radiation-Induced Parotid Gland Injury in Rats. Dose Response.

[61] Alaofi A.L. (2020). Sinapic Acid Ameliorates the Progression of Streptozotocin (STZ)-Induced Diabetic Nephropathy in Rats via NRF2/HO-1 Mediated Pathways. Front. Pharmacol..

[62] Raish M., Shahid M., Bin Jardan Y.A., Ansari M.A., Alkharfy K.M., Ahad A., Abdelrahman I.A., Ahmad A., Al-Jenoobi F.I. (2021). Gastroprotective Effect of Sinapic Acid on Ethanol-Induced Gastric Ulcers in Rats: Involvement of Nrf2/HO-1 and NF-κB Signaling and Antiapoptotic Role. Front. Pharmacol..

[63] Demir M., Altındağ F. (2022). Sinapic Acid Ameliorates Cisplatin-Induced Disruptions in Testicular Steroidogenesis and Spermatogenesis by Modulating Androgen Receptor, Proliferating Cell Nuclear Antigen and Apoptosis in Male Rats. Andrologia.

[64] Badawi M.S. (2022). A Study on the Antioxidant Activity of Rosmarinic Acid Against Carbon Tetrachloride-Induced Liver Toxicity in Adult Male Albino Rats. Int. J. Morphol..

[65] Ahmad Ansari M., Shahid M., Ahmad S.F., Ahmad A., Alanazi A., Malik A., Bin Jardan Y.A., Attia S.M., Bakheet S.A., Raish M. (2023). Sinapic Acid Alleviates 5-Fluorouracil-Induced Nephrotoxicity in Rats via Nrf2/HO-1 Signalling. Saudi Pharm. J..

[66] Martin K.R., Appel C.L. (2010). Polyphenols as Dietary Supplements: A Double-Edged Sword. Nutrition and Dietary Supplements.

[67] Granato D., Mocan A., Câmara J.S. (2020). Is a Higher Ingestion of Phenolic Compounds the Best Dietary Strategy? A Scientific Opinion on the Deleterious Effects of Polyphenols in Vivo. Trends Food Sci. Technol..

[68] Kim H.J., Bae Y.C., Park R.W., Choi S.W., Cho S.H., Choi Y.S., Lee W.J. (2002). Bone-Protecting Effect of Safflower Seeds in Ovariectomized Rats. Calcif. Tissue Int..

[69] Sengupta P. (2013). The Laboratory Rat: Relating Its Age with Human’s. Int. J. Prev. Med..

[70] Folwarczna J., Mierzwa K., Stolarczyk K., Zych M., Śliwiński L., Burczyk J., Trzeciak H.I. Wpływ Kwasu Ferulowego Na Układ Kostny w Eksperymentalnej Osteoporozie Wywołanej Niedoborem Estrogenów u Samic Szczurów. Proceedings of the 20 Naukowy Zjazd Polskiego Towarzystwa Farmaceutycznego—“Farmacja XXI wieku—Wyzwania i nadzieje”.

[71] Zych M., Wojnar W., Dudek S., Kaczmarczyk-Sedlak I. (2019). Rosmarinic and Sinapic Acids May Increase the Content of Reduced Glutathione in the Lenses of Estrogen-Deficient Rats. Nutrients.

[72] Liguori I., Russo G., Curcio F., Bulli G., Aran L., Della-Morte D., Gargiulo G., Testa G., Cacciatore F., Bonaduce D. (2018). Oxidative Stress, Aging, and Diseases. Clin. Interv. Aging.

[73] Chen C. (2016). Sinapic Acid and Its Derivatives as Medicine in Oxidative Stress-Induced Diseases and Aging. Oxid. Med. Cell. Longev..

[74] Zhao J., Xu L., Jin D., Xin Y., Tian L., Wang T., Zhao D., Wang Z., Wang J. (2022). Rosmarinic Acid and Related Dietary Supplements: Potential Applications in the Prevention and Treatment of Cancer. Biomolecules.

