# Supplementary material for: Effects of Rosmarinic Acid and Sinapic Acid on the Skeletal System in Ovariectomized Rats
Source: Nutrients. 2026 Jan 18;18(2):301. doi: 10.3390/nu18020301 (PMC12845317; doi:10.3390/nu18020301)
Supplement: Supplementary file 1 [file nutrients-18-00301-s001.zip › nutrients-4026666-supplementary.pdf]

Supplementary materials

Table S1. Effects of rosmarinic acid and sinapic acid administered orally for four weeks on L4 vertebra mass, composition, mineralization and density in rats with estrogen deficiency.

Parameter/Group	Sham-Operated Control Rats	Ovariectomized Control Rats	OVX+				
			E	RA10	RA50	SA5	SA25
Bone mass (g)	0.198 ± 0.022	0.198 ± 0.018	0.202 ± 0.015	0.198 ± 0.018	0.198 ± 0.017	0.191 ± 0.028	0.200 ± 0.013
Bone mineral mass (g)	0.085 ± 0.009	0.084 ± 0.006	0.087 ± 0.007	0.083 ± 0.007	0.084 ± 0.008	0.082 ± 0.012	0.085 ± 0.006
Mass of bone mineral/ bone mass ratio	0.430 ± 0.022	0.427 ± 0.024	0.431 ± 0.10	0.422 ± 0.014	0.422 ± 0.010	0.428 ± 0.009	0.423 ± 0.017
Mass of bone water/ bone mass ratio	0.334 ± 0.015	0.346 ± 0.019	0.335 ± 0.016	0.347 ± 0.019	0.349 ± 0.013	0.339 ± 0.017	0.344 ± 0.019
Mass of bone organic substances/bone mass ratio	0.236 ± 0.015	0.228 ± 0.014	0.234 ± 0.010	0.231 ± 0.007	0.229 ± 0.005	0.233 ± 0.009	0.234 ± 0.005
Calcium content (g/g of bone mineral)	0.400 ± 0.004	0.405 ± 0.022	0.403 ± 0.007	0.401 ± 0.006	0.406 ± 0.007	0.408 ± 0.009	0.405 ± 0.008
Phosphorus content (g/g of bone mineral)	0.157 ± 0.003	0.157 ± 0.007	0.157 ± 0.002	0.156 ± 0.002	0.158 ± 0.002	0.159 ± 0.003	0.158 ± 0.002
Magnesium content (g/g of bone mineral)	0.010 ± 0.002	0.011 ± 0.002	0.011 ± 0.002	0.012 ± 0.002	0.011 ± 0.003	0.010 ± 0.003	0.009 ± 0.002
Bone density (g/cm^3)	1.572 ± 0.054	1.520 ± 0.040 *	1.542 ± 0.013 #	1.525 ± 0.024 *	1.525 ± 0.018 *	1.528 ± 0.024 *	1.509 ± 0.060 *
Bone mineral density (g/cm^3)	0.676 ± 0.052	0.648 ± 0.039	0.664 ± 0.016	0.643 ± 0.030	0.644 ± 0.023	0.653 ± 0.021	0.638 ± 0.046

The results are presented as means ± standard deviation (SD; *n* = 10). OVX+E—bilaterally ovariectomized rats administered with estradiol at a dose of 0.2 mg/kg; OVX+RA10—bilaterally ovariectomized rats administered with rosmarinic acid at a dose of 10 mg/kg; OVX+RA50—bilaterally ovariectomized rats administered with rosmarinic acid at a dose of 50 mg/kg; OVX+SA5—bilaterally ovariectomized rats administered with sinapic acid at a dose of 5 mg/kg; OVX+SA25—bilaterally ovariectomized rats administered with sinapic acid at a dose of 25 mg/kg. The Kruskal–Wallis test followed by the Mann–Whitney U test was used for statistical evaluation of the significance of the results. * $p \leq 0.05$ – in comparison with the sham-operated control rats (SHAM group). # $p \leq 0.05$ in comparison with the OVX control rats (OVX group).

Table S2. Effects of rosmarinic acid and sinapic acid administered orally for four weeks on value of maximum load in the femoral neck in rats with estrogen deficiency.

Parameter/Group	Sham-Operated Control rats	Ovariectomized Control Rats	OVX+				
			E	RA10	RA50	SA5	SA25
Maximum load in the femoral neck (N)	86.9 ± 10.7	87.1 ± 15.5	89.4 ± 9.8	82.6 ± 12.0	84.2 ± 11.4	81.0 ± 9.1	81.7 ± 8.4

The results are presented as means ± standard deviation (SD; *n* = 10). OVX+E—bilaterally ovariectomized rats administered with estradiol at a dose of 0.2 mg/kg; OVX+RA10—bilaterally ovariectomized rats administered with rosmarinic acid at a dose of 10 mg/kg; OVX+RA50—bilaterally ovariectomized rats administered with rosmarinic acid at a dose of 50 mg/kg; OVX+SA5—bilaterally ovariectomized rats administered with sinapic acid at a dose of 5 mg/kg; OVX+SA25—bilaterally ovariectomized rats administered with sinapic acid at a dose of 25 mg/kg. The Kruskal–Wallis test followed by the Mann–Whitney U test was used for statistical evaluation of the significance of the results.
